# Effects of Electrical Stimulation on the Signal Transduction-Related Proteins, c-Src and Focal Adhesion Kinase, in Fibroblasts

**DOI:** 10.3390/life12040531

**Published:** 2022-04-04

**Authors:** Kazuo Katoh

**Affiliations:** Laboratory of Human Anatomy and Cell Biology, Faculty of Health Sciences, Tsukuba University of Technology, Tsukuba 305-8521, Japan; katoichi@k.tsukuba-tech.ac.jp; Tel.: +81-29-858-9557

**Keywords:** cytoskeleton, electrical stimulation, focal adhesion kinase, c-Src

## Abstract

Electrical stimulation of the skin and muscles, e.g., in the fields of rehabilitation medicine and acupuncture, is known to locally increase blood flow and metabolism, and thus have beneficial health effects. However, little is known about the changes in cellular morphology or regulation of the localization of specific proteins in response to electrical stimuli. The present study was performed to examine the effects of electrical stimulation on the cytoskeletal system of cultured fibroblasts. Following application of electrical stimulation to cultured fibroblastic cells for a period of about 2 h, the stress fibers in the cells became thicker and the cells showed a contracted appearance. Cells were subjected to periodic electrical stimulation for 0 (unstimulated control), 2, 5, or 20 h. The stress fibers showed an increase in thickness within 2 h, and became gradually thicker until 20 h. In addition, the focal adhesions and stress fibers were enlarged after 2 h of continuous stimulation, and both stress fibers and focal adhesions became larger and thicker after 20 h of periodic stimulation. Cells showed increased staining of focal adhesions with anti-phosphotyrosine antibody (PY-20) after electrical stimulation. Cells also showed increased staining of tyrosine-phosphorylated focal adhesion kinase (FAK) (pY397) and tyrosine-phosphorylated c-Src (pY418), indicating that electrical stimulation affected signal transduction-related proteins.

## 1. Introduction

In the fields of rehabilitation medicine and acupuncture, electrical stimulation of the skin and muscles has been shown to locally increase blood flow and metabolism, and thus to have beneficial health effects. Electrical stimulation has been shown to have similar effects as mechanical stimuli in many cell types. The mechanical stimulation of vascular endothelial cells by blood flow was shown previously to cause marked increases in the level of endothelial cellular Src (c-Src), an intracellular signaling protein, as well as increases in the number and size of focal adhesions [1]. Activated phosphotyrosine-containing proteins are particularly abundant in focal adhesions, and c-Src is colocalized with focal adhesion kinase (FAK) in these areas of the cell [2]. Signal transduction-related proteins, including FAK, c-Src, and Rho A, have been reported to undergo tyrosine phosphorylation, and to be colocalized with the constituent proteins of stress fibers and focal adhesions [3,4,5]. This study was performed to examine the direct effects of electrical stimulation on various cell types to clarify the effects of electrotherapy by acupuncture and rehabilitation treatment on signal transduction-related proteins.

Several studies involving electrical stimulation of cells in culture, which were mainly related to effects on cell motility and cell adhesion, have been reported to date. There have been many studies of the morphology of stress fibers and focal adhesions as well as the dynamics of their constituent proteins under normal culture conditions. However, to date, there have been few studies regarding the states of cells and activation of proteins during the application of electrical stimulation to living cells in culture. Stress fibers and focal adhesions may have roles in the regulation of cell morphology, movement, and contraction, and in signaling between the cell and the external environment [3,4,6,7]. Cells undergo reconstruction of their cytoskeleton in response to mechanical signals. For example, vascular endothelial cells show changes in their cytoskeleton and cell morphology in response to mechanical signals, such as shear stress and stretch tension caused by blood flow and blood pressure, contributing to homeostasis of the intravascular environment [8]. It is also well known that epithelial cells show changes in their cytoskeleton and cell adhesion depending on the stiffness of the extracellular matrix, thus regulating motility, proliferation, differentiation, and cell death [9]. Mechanoreceptor channels on the cell membranes are known to act as mechanosensors or molecules that accept and transmit mechanical signals, and detailed analyses of bacterial mechanoreceptor channels, including their conformational changes upon mechanical stimulation, have been reported [10,11]. It has also been suggested that c-Src may be involved in the reception of mechanical stimulation of vascular endothelial cells by blood flow in the guinea pig aorta, and the activation of c-Src may be related to vascular lesions, such as atherosclerosis [1]. Although there have been many studies on c-Src, these have mainly been conducted using biochemical techniques. There have been no previous reports of the activation of tyrosine phosphorylation-activated proteins, such as c-Src and FAK, by electrical stimulation. In addition, there have been no studies regarding the responses of individual cells to electrical stimulation, and the mechanisms underlying these responses have not been elucidated. The present study was performed to examine the tyrosine phosphorylation of proteins related to signal transduction and the effects of acupuncture stimulation in individual cells.

For example, acupuncture applies mechanical and electrical stimuli to various cells in the body via the inserted needles. The inserted needles are either mechanically stimulated or penetrate the cell membrane, resulting in a change in membrane potential. Furthermore, additional electrical stimulation using the acupuncture needles as electrodes is thought to cause a potential difference, which stimulates the cells. Such electrical stimuli, which may be uniform or pulsed, stimulate blood flow, suppress sympathetic nerve activity, and ameliorate muscle stiffness. Thus, such electrical stimulation has some effects on the body and in regulating the normal functioning of cells, which are thought to be due to the activation of certain proteins within the cells. However, it remains unclear which structures and proteins within the cells are actually affected by electrical stimulation, and the mechanisms underlying the changes in individual cells have yet to be elucidated. Using typical connective tissue cells (NIH 3T3 fibroblastic cells), this study was performed to examine which proteins in the cells are affected by electrical stimulation, the mechanisms by which these proteins cause cellular changes and normalization of cellular functions, and to determine whether the activation of tyrosine-phosphorylated proteins by electroacupuncture enhances the cytoskeletal system.

Electrical stimulation involves the stimulation of certain types of cells with an electric current, which is thought to stimulate blood flow, suppress sympathetic nerve activity, and ameliorate muscle stiffness. This stimulation appears to have some effects on the cells, and to promote their normal functions by activation of proteins within the cells. However, it remains unclear which structures within the cells are actually affected by electrical stimulation and how the stimuli cause changes in individual cells. This study focused on identifying the proteins affected by electrical stimulation, and elucidation of the changes in tyrosine phosphorylation of the signal transduction proteins, FAK and c-Src, in response to stimulation responsible for changes in the cells.

## 2. Materials and Methods

### 2.1. Electrical Stimulation of the Cells

**Figure 1:**
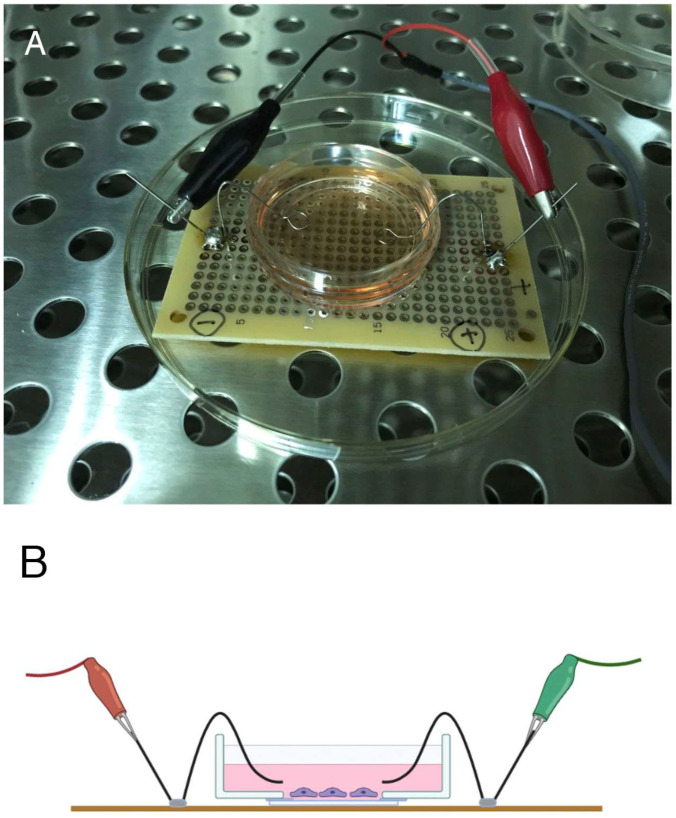
Apparatus for providing single-pulse electrical stimulation to cells and simple stimulation device. We developed a device using an electronic circuit and platinum wire to apply electrical stimulation to cultured cells, and confirmed that it could apply electrical stimulation to the cells (**A**). A schematic illustration is shown in (**B**).

This study was performed using cultured fibroblasts (3T3 cells; NIH, Bethesda, MD, USA). In vivo, fibroblasts are located in the skin and may receive electrical stimuli directly. Here, the cells were subjected to single-pulse electrical stimulation using an electrical stimulator (SEN-2201; Nihon Kohden, Tokyo, Japan), and the morphological changes in the cells were examined by fluorescence microscopy. The device used to apply the electrical stimulation to the cultured cells was made using a circuit board and pure (100%) platinum electrodes (Figure 1A,B).

The pattern and duration of electrical stimulation applied in this study were adjusted, as the growth environment in culture differs from that of cells in vivo. The details of signaling protein activation, changes in the cytoskeletal system, and changes in cell morphology in cells given electrical stimulation compared to unstimulated controls were examined. Activation of proteins by tyrosine phosphorylation was found to be prominent, and they showed marked accumulation in focal adhesions. Phosphotyrosine-containing proteins are thought to be involved in a wide range of intracellular signal transduction systems, and the intracellular skeletal structures of stress fibers and focal adhesions are expected to be involved in the transmission of various cellular stimuli as structures that connect the inside of the cell to the external environment. This study attempted to identify the intracellular signal transduction proteins that were suggested to be activated by electrical stimulation.

Based on the results of our preliminary research, the cells were stimulated at 50 V, 60 times/min, in a cyclic manner for 0 (unstimulated control), 2, 5, and 20 h [12,13]. The stimulation pulse was applied as a square wave with a duration of 2.0 ms. Electrolysis occurred from the positive to the negative electrode, both of which were 0.1 mm in diameter, made of 100% platinum, and the tip was bent into a loop with a diameter of 5–7 mm. One electrode was placed on each side of the culture dishes 35 mm in diameter (two electrodes in total) and pulsed electrical stimuli were applied. Fibroblasts located in the center of the glass-bottomed dishes where the electrical stimuli were applied were observed by epifluorescence microscopy. An electric field was generated between the positive (+) and negative (−) electrodes, but its distribution was unclear in this experiment. No changes were observed in the color of phenol red in the culture medium, so the pulse stimulus applied to the cells did not seem to affect the pH of the culture medium.

The conductivity of the culture medium in this study was determined by directly measuring the voltage and electrical current. The measured resistance of the culture medium used in this study was 324.68 ohm (50 volts: 0.154 A). Electric resistivity in the center of the cultured dish was measured as follows:

(1) $$\rho \;(\mathrm{electrical}\;\mathrm{resistivity})=\frac{RA}{L}$$

where *R* is the resistance (in ohm), *A* is the cross-sectional area of the center of the culture dish (in cm^2^), and *L* is the length between both electrodes (2 cm).

(2) $$\rho =\frac{324.68\times 1.75}{2}=284.1\;\mathrm{ohm}/\mathrm{cm}$$

(3) $$S/\mathrm{cm}=\frac{1}{284.1}=0.0035$$

0.0035 S/cm = 0.35 S/m(4)

The measured conductivity of the medium was 0.35 S/m. A current of 0.154 A was delivered to the cultured cells through the culture medium.

Morphological changes in the cells and in phosphotyrosine-containing proteins were analyzed following exposure to electrical stimulation for 2, 5, or 20 h, and the results were compared with unstimulated controls. In particular, the intracellular skeletal structures, such as stress fibers and focal adhesions, are expected to be involved in the transmission of various cellular stimuli that connect the external environment to the inside of the cell. Based on the hypothesis that stress fibers and focal adhesions function as receptors for various stimuli [14,15,16], we focused on the localization and morphological changes of the component proteins of these structures associated with electrical stimulation.

### 2.2. Cell Culture

Fibroblasts (3T3 cells; NIH) were cultured in a 1:1 mixture of Dulbecco’s modified Eagle’s medium and nutrient mixture (DMEM/F-12; Gibco, Grand Island, NY, USA), pH 7.4, containing 50 units/mL of penicillin, 50 µg/mL of streptomycin, and 10% fetal bovine serum (Gibco). The cells were maintained at 37 °C in a humidified, 5% CO_2_ atmosphere. Cells were cultured on glass-bottomed culture dishes 35 mm in diameter (Matsunami Glass, Tokyo, Japan) overnight and used in the experiments.

Normal fibroblasts were transfected with a vector encoding enhanced green fluorescent protein (eGFP)-conjugated paxillin (eGFP-paxillin) as a focal adhesion marker using Tfx-50 reagent (Promega, Madison, WI, USA). Stably transfected cells were selected with G418 (Wako Chemical, Tokyo, Japan), and stored in liquid nitrogen. eGFP-paxillin expression was found in all cells, indicating that it can be used as a marker of focal adhesions. The size and shape of focal adhesions of eGFP-paxillin-transfected cells were the same as those of focal adhesions stained with anti-paxillin antibodies, and can be used as a marker of the focal adhesions for the double labeling together with monoclonal antibodies. Photographs were taken with a combination of 60× PlanApo lens (Olympus, Tokyo, Japan) and CoolSnap EZ CCD camera (Roper Scientific, Tucson, AZ, USA), and all fluorescence images were taken at the same magnification. The contrast and brightness on the images were also adjusted to the same level using ImageJ image analysis software (NIH).

### 2.3. Immunofluorescence Microscopy

Monoclonal anti-paxillin (BD Biosciences, Franklin Lakes, NJ, USA), monoclonal anti-phosphotyrosine antibodies (PY-20; Transduction Laboratories, Lexington, KY, USA), polyclonal anti-phospho-c-Src (pY418) (BioSource, San Jose, CA, USA), and polyclonal anti-phospho-FAK (pY397) antibodies (BioSource) were purchased from the sources shown. FITC-labeled phalloidin for staining of actin filaments was also purchased from Cytoskeleton (Denver, CO, USA). FITC (Cytoskeleton) and rhodamine-labeled phalloidin (Molecular Probes, Eugene, OR, USA) for staining of actin filaments were also purchased from the sources shown.

Normal or GFP-paxillin-transfected (as a marker for focal adhesions) fibroblasts were fixed with 1% paraformaldehyde in phosphate-buffered saline (PBS) for 30 min. GFP-paxillin-transfected cells were directly observed by epifluorescence microscopy (Olympus, Tokyo, Japan). Normal cells were permeabilized by treatment with 0.05% Triton X-100 in PBS for 5 min. The cells were treated with 10% normal goat serum for 30 min at room temperature and then stained with anti-paxillin, anti-PY-20, polyclonal anti-phospho-c-Src (pY418), and polyclonal anti-phospho-FAK (pY397) antibodies. After washing in PBS for 20 min, the fixed specimens were incubated with fluorescein-conjugated anti-mouse IgG or anti-rabbit IgG. Samples were then observed by conventional epifluorescence microscopy. More than 10 experiments were performed for each electrical stimulation period and the results were compared by epifluorescence microscopy.

### 2.4. ELISA

The STAR FAK [pY397] and Src [pY418] ELISA Kits (Millipore, Darmstadt, Germany) are solid-phase sandwich enzyme-linked immunosorbent assay (ELISA) kits. This assay was designed to detect and quantify the levels of mouse FAK phosphorylated at tyrosine residue 397 [FAK (pY397)] or c-Src phosphorylated at tyrosine residue 418 [Src (pY418)] in cell extracts. The optical densities of the plates at 450 nm (OD_450_) were read using an Elx808 Microplate Reader (Lonza, Basel, Switzerland). Cells were cultured on 5 cm culture dishes and stimulated for 0 (unstimulated control), 2, 5, or 20 h to induce electrical activation, as described above. The culture medium was removed, and cells were washed twice with ice-cold PBS. PBS was removed and cells were incubated with 0.5 mL of RIPA buffer containing protease inhibitors (Takara, Ohtsu, Japan). Cells were then scraped off the plates with a rubber policeman. Cells in RIPA buffer were transferred to microfuge tubes, incubated on ice for 15 min, centrifuged at 12,000 rpm for 5 min at 4 °C, and then used for ELISAs. Four or five experiments were performed for each condition, and the means ± SEM were calculated with Microsoft Excel (Microsoft, Redmond, WA, USA). The means of two groups were compared using the unpaired two-tailed Student’s *t*-test. In all analyses, *p* < 0.05 was taken to indicate statistical significance. 

### 2.5. Quantitative Analysis of Focal Adhesion Number and Area

Quantitative analysis was performed using the open source Fiji image analysis software [17]. The sizes of focal adhesions that had been electrically stimulated for 0 (unstimulated control), 2, 5, and 20 h were measured using Fiji in GFP-paxillin-transfected fibroblasts before (Control 0 h) and after electrical stimulation. Unstimulated control of cultured fibroblasts without electrical stimulation for 20 h was also measured (Control 20 h). Images of focal adhesions were converted from grayscale to binary images with white and black values. Focal adhesion number and area were counted using Fiji. Statistical analyses were performed using Microsoft Excel. The means of two groups were compared using the unpaired two-tailed Student’s *t*-test. In all analyses, *p* < 0.05 was taken to indicate statistical significance.

## 3. Results

Mouse 3T3 fibroblasts were stimulated with a single-pulse current applied using an electrical stimulator (SEN-2201; Nihon Kohden). After stimulation, the morphology of the fibroblasts, the cytoskeletal proteins in the cells, and the activation of the signaling proteins were examined in detail. In particular, c-Src and FAK activation were found to play important roles in cell motility and adhesion, suggesting that changes in the activation of signaling proteins in single cells are important for the response to electrical stimulation. c-Src and FAK activation are components of an intracellular and extracellular signaling pathway that may function as a signal transduction system to induce cell adhesion and changes in cell morphology. This study sought to clarify the mechanisms underlying activation of cells by electrical stimulation.

Figure 1A,B shows the electrical stimulator used to apply electrical stimuli to cultured cells. Preliminary experiments indicated that cyclic stimulation at 50 V, 60 times/min (1 Hz) for 2.0 ms as a square wave pulse resulted in changes in cell shape and morphology within 2 h. These morphological changes in the cells and changes in phosphotyrosine-containing proteins were analyzed in cells exposed to electrical stimulation. The platinum wire connected to the electrical stimulator was immersed in the culture medium on both sides of the culture dish without coming into direct contact with the cells, 1–2 mm above the fibroblasts. 

**Figure 2:**
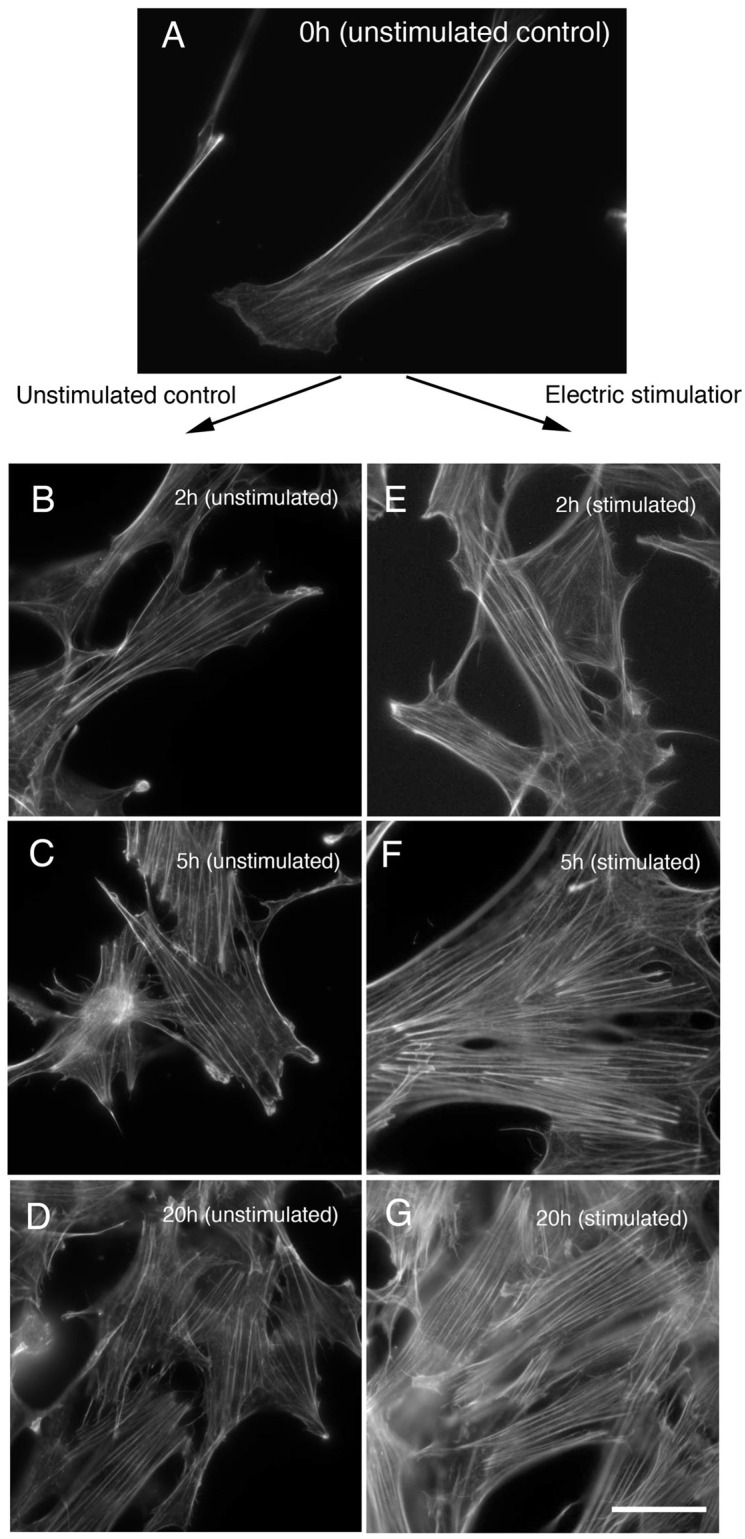
Changes in stress fibers during electrical stimulation of cells. The stress fibers of fibroblasts were significantly altered with the application of periodic electrical stimuli at 50 V, 60 times/min. Electrical stimuli were applied to the cells for 0 (control) (**A**), 2 (**E**), 5 (**F**), or 20 h (overnight) (**G**). The stress fibers began to increase in number after about 1 h of electrical stimulation. The stress fibers became remarkably thicker after about 2 h of electrical stimulation (compare (**B**) unstimulated control, and (**E**) electric stimulation for 2 h). The stress fibers became more thicker after about 5 h of electrical stimulation (compare (**C**) unstimulated control, and (**F**) electric stimulation for 5 h). After 20 h of periodic electrical stimulation, the number of stress fibers remained the same, but the fibers increased in thickness (compare (**D**) unstimulated control for 20 h, and (**G**) electric stimulation for 20 h). Bars: 20 μm.

Figure 2A shows unstimulated control of cultured fibroblast stained with rhodamine-labeled phalloidin for the stress fiber detection. The stress fibers began to enlarge in size after 1 h of electrical stimulation, and then became thicker after about 2 h (Figure 2E). After 2–5 h of electrical stimulation (Figure 2E,F), the stress fibers were thicker and larger than in unstimulated controls (Figure 2B,C). After 5 h of electrical stimulation, stress fibers gradually increased in size and thickness (Figure 2F). After 20 h of electrical stimulation, the stress fibers showed increased thickness, but their number remained almost the same as at 2 h in a cell (compare Figure 2G for electrical stimulation and Figure 2D for unstimulated control for 20 h). 

**Figure 3:**
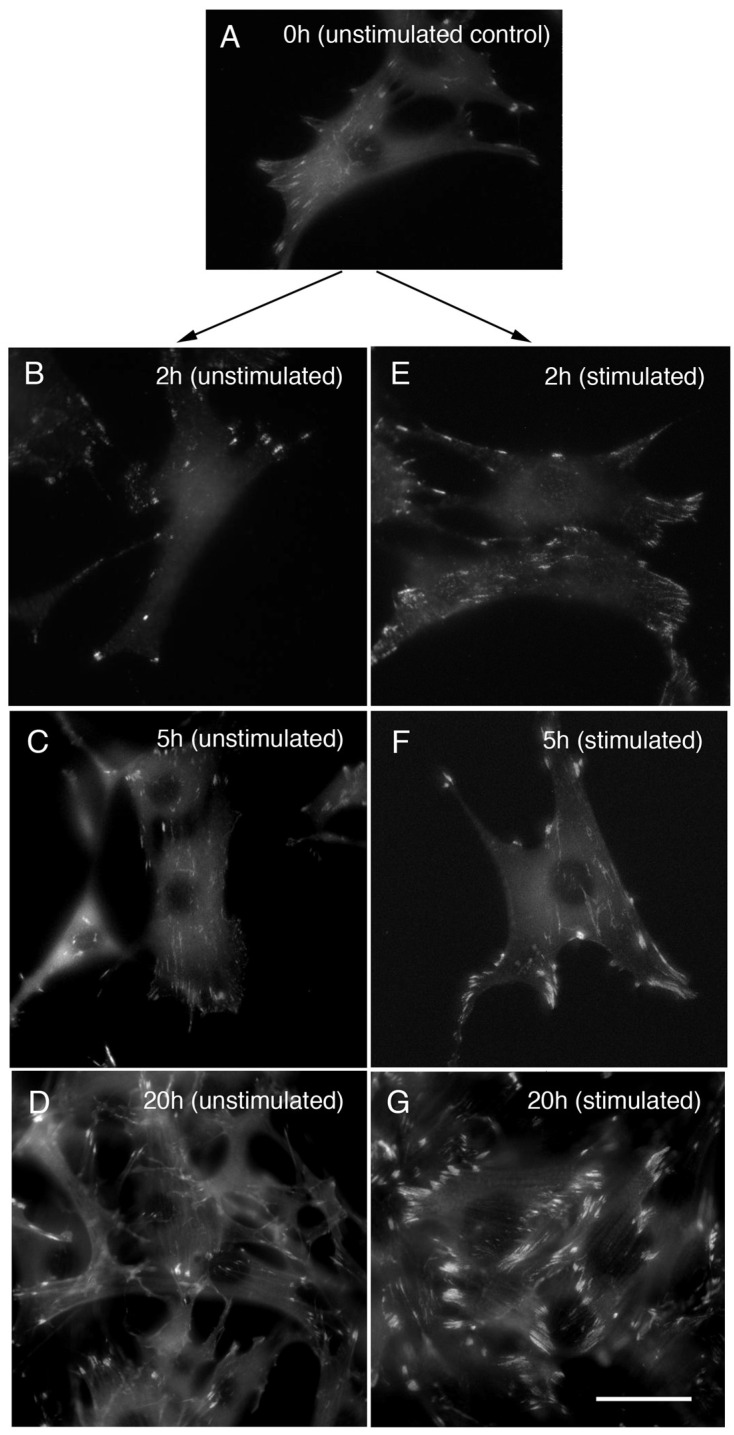
Changes in focal adhesions after electrical stimulation of cells. Fibroblasts were stained with monoclonal anti-paxillin antibody, and the focal adhesions were examined after periodic electrical stimulation (0, 2, 5, 20 h). Focal adhesions are gradually enlarged in size, according to the electrical stimulation for 2 (**E**), 5 (**F**), and 20 (**G**) hours. (**A**) 0 h (unstimulated control); (**B**) 2 h (unstimulated control); (**C**) 5 h (unstimulated control); (**D**) 20 h (unstimulated control); (**E**) 2 h (electrical stimulation); (**F**) 5 h (electrical stimulation); (**G**) 20 h (electrical stimulation). Bars: 20 μm.

Next, the changes in focal adhesions after electrical stimulation were examined in cells stained with monoclonal anti-paxillin antibody and stimulated for 0 (unstimulated control; Figure 3A), 2, 5, or 20 h (Figure 3E–G). Unstimulated controls of cultured fibroblasts for 2, 5, 20 h were also shown (Figure 3B–D). The focal adhesions gradually became larger after 2, 5, or 20 h (Figure 3E–G) of electrical stimulation compared with unstimulated controls (Figure 3B–D). After 2 h of electrical stimulation, both focal adhesions (Figure 3E–G) and stress fibers (Figure 2E–G) were shown to have begun to increase in number and size. Both stress fibers and focal adhesions were thicker and after 20 h of periodic electrical stimulation compared to unstimulated controls (compare Figure 2G for the stress fibers and Figure 3G for the focal adhesions).

**Figure 4:**
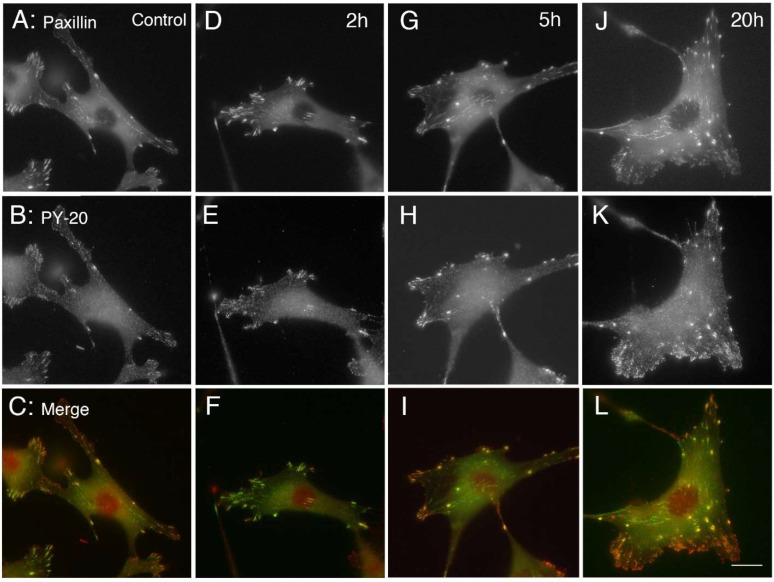
Increase in focal adhesions and accumulation of phosphotyrosine-containing proteins by electrical stimulation. After 2 h of electrical stimulation of GFP-paxillin-transfected cells, the focal adhesions began to enlarge (**D**–**F**). After 5 (**G**–**I**) to 20 h (**J**–**L**) of periodic electrical stimulation, the focal adhesions showed intense staining with monoclonal anti-phosphotyrosine antibody (PY-20). (**A**–**C**) Control (no electrical stimulation, 0 h). (**A**) GFP-paxillin. (**B**) Anti-phosphotyrosine antibody (PY-20). (**C**) Merge. (**D**–**F**) 2 h of electrical stimulation. (**D**) GFP-paxillin. (**E**) Anti-phosphotyrosine antibody (PY-20). (**F**) Merge. (**G**–**I**): 5 h of electrical stimulation. (**G**) GFP-paxillin. (**H**) Anti-phosphotyrosine antibody (PY-20). (**I**) Merge. (**J**–**L**) 20 h of electrical stimulation. (**J**) GFP-paxillin. (**K**) Anti-phosphotyrosine antibody (PY-20). (**L**) Merge (PY-20 staining, red; eGFP-paxillin, green). Bars: 20 μm.

Figure 4 shows the increases in size of focal adhesions and accumulation of phosphotyrosine-containing proteins by electrical stimulation. Unstimulated control for 0 h is shown in Figure 4A–C. After 2 h of electrical stimulation of eGFP-paxillin-transfected cells, the focal adhesions began to increase in size after 2 h (Figure 4D), and gradually enlarged the size of focal adhesions after 5 h (Figure 4G) and 20 h (Figure 4J), and showed intense staining with anti-phosphotyrosine antibody (PY-20) after 2 h (Figure 4E), 5 h (Figure 4H), and 20 h (Figure K). Merged images of both eGFP-paxillin and anti-phosphotyrosine (PY-20) are shown in Figure 4C,F,I,L. Tyrosine-phosphorylated proteins were accumulated in focal adhesions revealed by eGFP-paxillin.

**Figure 5:**
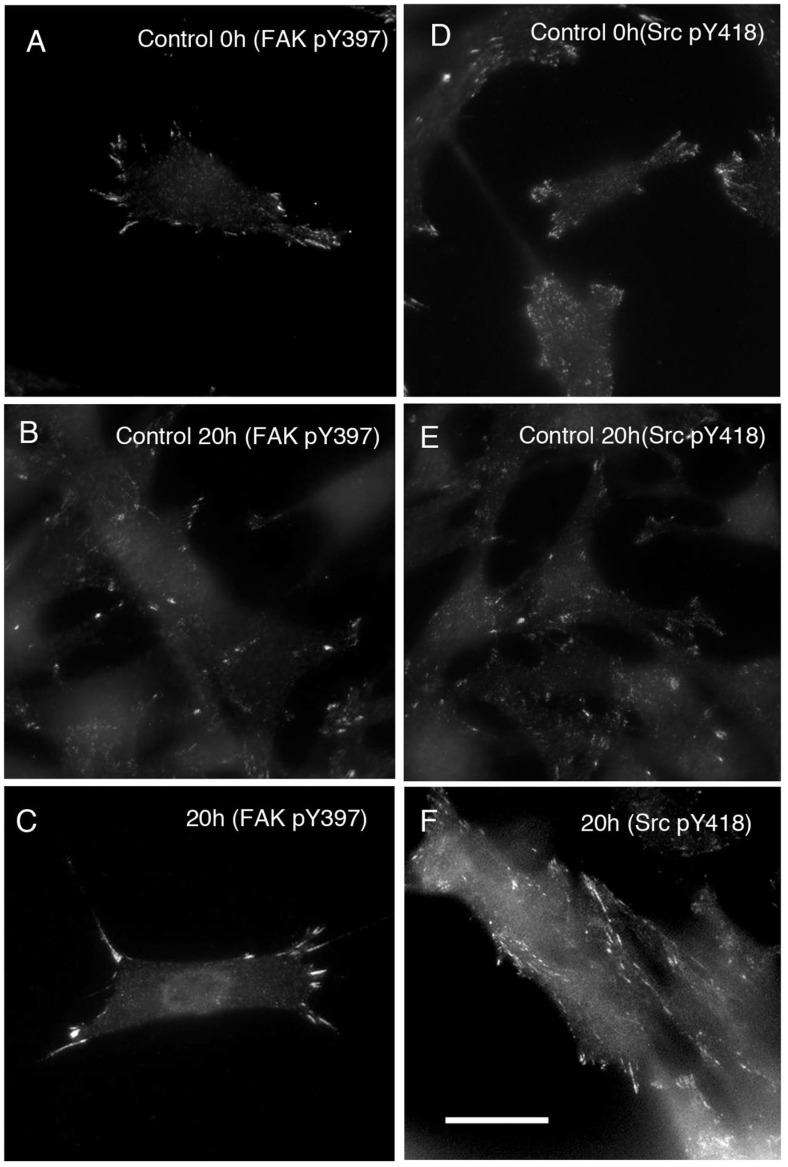
Staining of the active forms of FAK and c-Src in cells with periodic electrical stimulation. Cells stimulated for 0 (unstimulated control) (**A**,**D**), 20 (unstimulated control for 20 h) (**B**,**D**), and 20 h (**C**,**F**) were stained with anti-phospho-FAK (pY397) antibody (**A**–**C**) or anti-phospho-c-Src (pY418) antibody (**D**–**F**). Intense staining for both tyrosine-phosphorylated FAK and c-Src was observed in focal adhesions after 20 h electrical stimulation (**C**,**F**). Unstimulated control stained with anti-phospho-FAK (pY397) antibody (**A**) or unstimulated control stained with anti-phospho-c-Src (pY418) antibody (**D**). Unstimulated control cultured for 20 h stained with anti-phospho-FAK (pY397) antibody (**B**) or unstimulated control cultured for 20 h stained with anti-phospho-c-Src (pY418) antibody (**E**). Electrical stimulation for 20 h stained with anti-phospho-FAK (pY397) antibody (**C**) or electrical stimulation for 20 h stained with anti-phospho-c-Src (pY418) antibody (**F**). Bars: 20 μm.

Figure 5 shows the results of staining for the active forms of FAK (pY397) and c-Src (pY418) in cells with periodic electrical stimulation. Cells stimulated for 20 h showed intense staining with anti-phospho-FAK (pY397) and anti-phospho-c-Src (pY418) antibodies in focal adhesions (Figure 5C,F, respectively).

**Figure 6:**
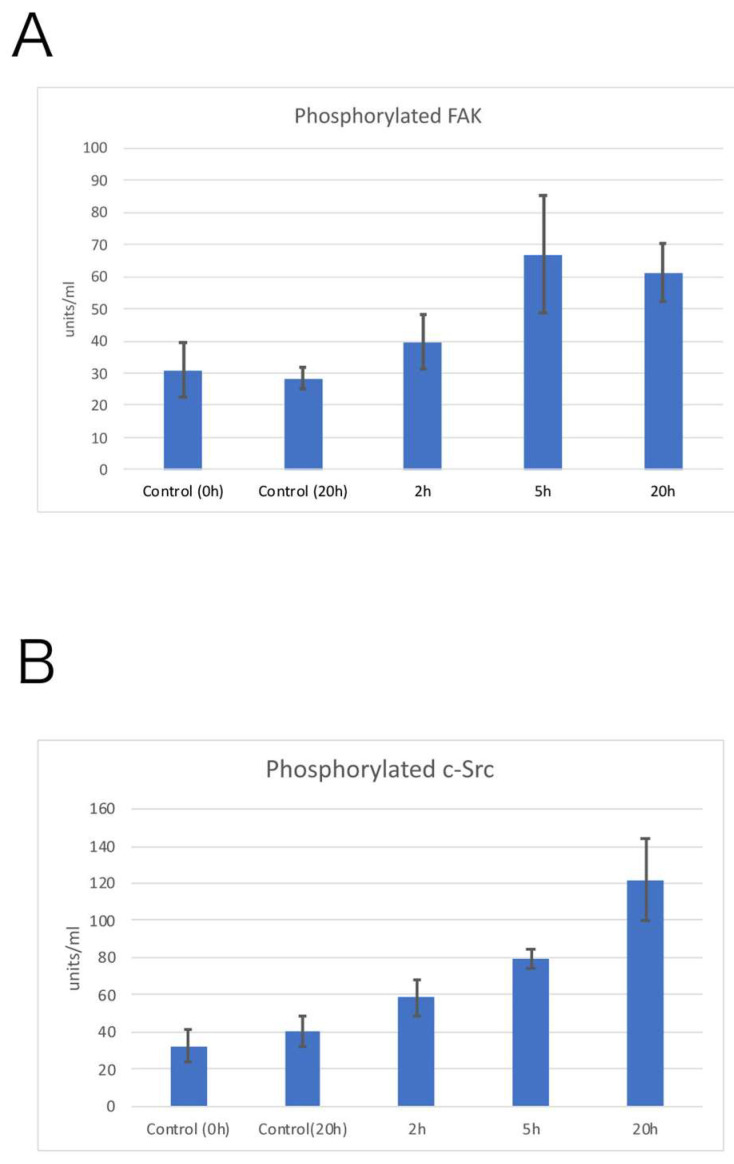
Changes in phosphorylated proteins, tyrosine-phosphorylated FAK (**A**), and tyrosine-phosphorylated c-Src (**B**) were examined by ELISA. Both the tyrosine-phosphorylated FAK (**A**) and tyrosine-phosphorylated c-Src (**B**) levels increased gradually according to the electrical stimulation time. The level of FAK phosphorylation remained almost the same between 5 and 20 h of electrical stimulation (**A**). The level of tyrosine-phosphorylated c-Src increased by about 3.7-fold compared to controls at 20 h of electrical stimulation (**B**).

The level of tyrosine-phosphorylated FAK and tyrosine-phosphorylated c-Src was examined by ELISA experiments. The level of tyrosine-phosphorylated FAK was increased by about twofold at 5 h compared to controls and remained almost constant from 5 to 20 h (Figure 6A), while the level of tyrosine-phosphorylated c-Src was increased by about 3.7-fold compared to the control level at 20 h (Figure 6B). The level of tyrosine-phosphorylated FAK relative to the control (0 h; no electrical stimulation) was increased at 2 h, and maximum phosphorylation was detected after 5 h of electrical stimulation. On the other hand, the level of tyrosine-phosphorylated c-Src was increased after 2 h of electrical stimulation and showed a gradual linear increase until 20 h. These results indicate that electrical stimulation increased the level of tyrosine phosphorylation of both FAK and c-Src, reflecting the microscopic results. Phosphorylation levels of the unstimulated control (Control 0 h) or 20 h (Control 20 h) in both tyrosine-phosphorylated FAK and tyrosine-phosphorylated c-Src were almost the same. 

**Figure 7:**
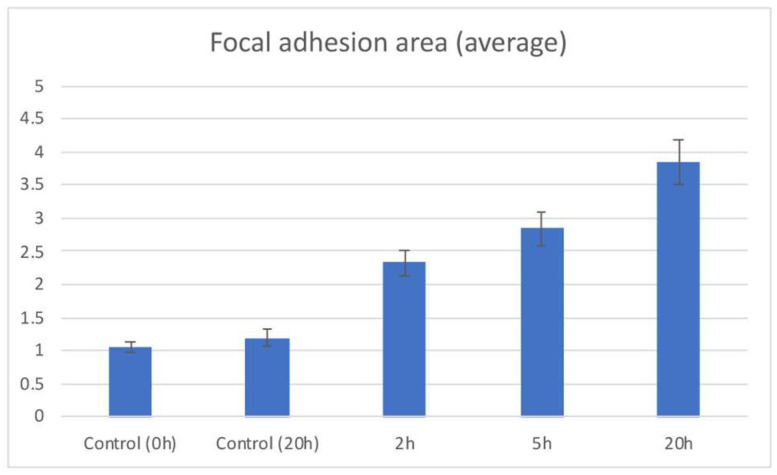
Quantification of focal adhesion area. The mean areas of focal adhesions are shown for the unstimulated control (Control 0 h), unstimulated control without electrical stimulation for 20 h (Control 20 h) 2, 5, and 20 h of electrical stimulation. Number of vertical bars is area of the focal adhesions (μm^2^). Error bars represent the SEM.

A graph of the average area of focal adhesions of fibroblasts transfected with GFP-paxillin is presented in Figure 7. The mean ± SEM areas of focal adhesions were 1.06 ± 0.08 µm^2^ (*n* = 109) for the unstimulated control, 2.33 ± 0.19 µm^2^ (*n* = 148) for 2 h of electrical stimulation, 2.84 ± 0.26 µm^2^ (*n* = 89) for 5 h of electrical stimulation, 3.841 ± 0.35 µm^2^ (*n* = 126) for 20 h of electrical stimulation, and 1.186 ± 0.12 µm^2^ (*n* = 104) for 20 h without electrical stimulation. These quantitative analyses strongly suggest that electrical stimulation causes enlargement of focal adhesions.

## 4. Discussion

Focal adhesions are structures that penetrate the cell membrane and serve as a connection between the inside of the cell and its substrate (glass or plastic surface in the case of cultured cells). The focal adhesions are directly connected to stress fibers on the inside of the cell, and can transmit signals, such as electrical stimuli, from the external environment into the cell, and are therefore considered to be signaling sites [5,14,18]. Therefore, it is likely that the focal adhesions and their associated contractile structures, the stress fibers, play important roles as receptors for electrical stimuli in cells cultured in the laboratory. The results of this study demonstrate marked enhancement of focal adhesions by electrical stimulation. Specific focal adhesion-associated proteins, such as vinculin, paxillin, talin, alpha-actinin, and integrins, are accumulated in these regions in the cell, along with several signaling proteins, such as FAK, c-Src, and Rho A, in close association with stress fibers [3,4,5,6,14,19,20,21]. These observations strongly suggest that focal adhesions play roles in transmitting specific migration and polarization signals from the external environment to the inside of the cell. Focal adhesions, along with stress fibers, recognize the boundary between the cell membrane and the extracellular matrix (ECM), and also determine cell orientation and polarity. The results of the present study demonstrate that electrical stimulation applied to the cells causes enlargement of focal adhesions and thickens stress fibers, as well as activating signal transduction-related proteins, such as c-Src and FAK, localized at the focal adhesions. These observations suggest that c-Src and FAK play important roles in the responses of cells to electrical stimulation.

Phosphorylated FAK (pY397) and phosphorylated c-Src (pY418) were shown to be localized in focal adhesions [2]. The Src family tyrosine kinases (SFKs) are a family of membrane-associated, non-receptor-type protein tyrosine kinases that have a number of roles in cell–matrix and cell–cell adhesion and are found in endosomal vesicles. Src mediates signal transduction via a variety of receptors [22,23], and activated Src has been shown to induce cell transformation in vitro [24,25,26]. Phosphorylation of FAK at Y397 promotes the formation of the FAK–Src complex. Then, direct phosphorylation by FAK of Src Y418 causes activation of Src. For example, the expression and activity of Src are elevated in many human epithelial cancers [27]. The first 16 N-terminal amino acids of Src are required for membrane binding [28], with the subsequent 17–84 amino acids constituting a unique domain, which is followed by the SH3 and SH2 domains connected by a short linker, and another linker connects the SH2 domain to the kinase domain required for most biological functions of Src [28]. Tyr527 in chicken Src (or Tyr530 in human Src) undergoes inhibitory phosphorylation by the C-terminal Src kinase. In the inactive or “closed” form of Src, the SH2 domain interacts with pTyr527, placing the SH3 domain in the correct position to interact with the polyproline type II helix of the kinase-SH2 linker region, preventing the conformational change in the N-terminal domain of the kinase. Activation can occur by dephosphorylation or mutation of Tyr527 or by binding of the activating ligand to the SH2 or SH3 domains [24,25,29,30].

Physical stimuli from the extracellular environment are transmitted into the cell via cell adhesion molecules, which regulate cell proliferation and differentiation. This study was performed to investigate the effects of electrical stimulation on fibroblast growth and differentiation, and elucidate the details of the effects on FAK and c-Src as molecules involved in cell adhesion. Tyrosine phosphorylation is one of the most common modes of regulating protein functions. In most cases, the protein will switch between a phosphorylated (active) and a nonphosphorylated (inactive) form. In this study, the levels of tyrosine-phosphorylated FAK (pTyr397) and tyrosine-phosphorylated c-Src (pTyr418) were increased by about 2- and 3.7-fold compared to controls within 5 and 20 h of electrical stimulation, respectively. Electrical stimulation causes tyrosine phosphorylation in certain types of cells, and changes the morphology of cytoskeletal components, such as focal adhesions and stress fibers.

Integrins are involved in many cellular phenomena, including gene expression, cell proliferation, differentiation, and cell death [31,32]. Integrins, a family of transmembrane glycoproteins that form hexamers comprising α- and β-subunits, with 16 types of the former and eight types of the latter [31], play roles in connecting the ECM to the cytoskeleton and transmit physical signals from the environment to the inside of the cell to induce cellular responses [32,33]. In the cytoplasm, the β-subunit is more critical than the α-subunit because it is directly linked to the cytoskeleton. FAK is a non-receptor protein tyrosine kinase that is involved in signaling from integrin-enriched adhesion sites that mediate cell adhesion to the extracellular substrate. Promoted signaling has been reported to be involved in adhesion-dependent cell survival and is critical for efficient cell migration in response to growth factor receptor and integrin stimulation [34,35]. FAK expression is upregulated in human tumors and has been shown to be correlated with malignancy and infection [36,37]. The results of the present study demonstrate the activation of FAK and c-Src in electrically stimulated cells, indicating that electrical stimulation affects the activation of focal adhesion component proteins.

Signaling through cell adhesion molecules is one of the mechanisms by which mechanical stimulation by blood flow regulates cellular activities, such as cell proliferation and morphological changes [32,33,38]. In the present study, the levels of tyrosine phosphorylation of FAK and c-Src were increased in focal adhesions of electrically stimulated cells. The mechanism underlying the response of fibroblasts to electrical stimulation is thought to involve the transmission of information to the intracellular signaling system via these cell adhesion molecules.

Previous studies of cellular responses to physical stimuli have shown that pressure stimulation in chondrocytes [38] and shear stress loading in vascular endothelial cells [32] resulted in increased integrin expression, activated the MAPK intracellular signaling cascade, and suppressed cell death. It has also been reported that osteoblast differentiation and cell proliferation were inhibited by extracellular physical stimulation [39]. In addition, activation of p38, a molecule involved in the MAPK cascade [39,40], by elongation stimulation was shown to promote osteoblast differentiation [39], and the reduction of p38 activity by microgravity was reported to suppress osteoblast differentiation [40]. In muscle differentiation, activation of the MAPK cascade induces the expression of MyoD family members [41,42]. In fibroblasts, activation of intracellular signaling through cell adhesion factors by electrical stimulation may promote the expression of the Src family [43]. Despite its routine use in clinical practice as a treatment for muscle atrophy, much remains unclear about the cellular effects of electrical stimulation. In this study, we observed dynamic changes in cytoskeletal structures, such as focal adhesions and stress fibers, induced by electrical stimulation.

Molecular and cellular biological analyses showed that electrical stimulation of fibroblasts resulted in elevated tyrosine phosphorylation of the Src family and FAK, and increased the number and size of focal adhesions and stress fibers. These observations show that electrical stimulation activated the intracellular signaling pathways involving c-Src and FAK. The increased tyrosine phosphorylation of Src family members and FAK in focal adhesions observed in the present study suggest that cell–substrate communication via signaling-related proteins and cell adhesion molecules is important for the formation of cytoskeletal components. Further studies are required to examine the effects of electrical stimulation on cell–cell communication, including downstream intracellular signaling. To our knowledge, there have been no previous reports on the enhancement of stress fibers and enlargement of focal adhesions in fibroblasts by electrical stimulation. This study was conducted using fibroblasts, and previous studies showed that electrical stimulation of fibroblasts enhances their cytoskeletal structure, but this has not been confirmed in other cell types. We are currently planning further studies to investigate the mechanisms underlying the effects of electrical stimulation on intracellular signal transduction and cell contractility.

## 5. Conclusions

This study elucidated the morphological changes in cultured cells associated with the application of electrical stimulation. The cells showed thickening of the stress fibers after 2 h of electrical stimulation, confirming that the cells were contracting. After 20 h of periodic electrical stimulation, the number of stress fibers did not change, but their thickness increased. In addition, the focal adhesions became larger after 2 h of electrical stimulation, and both stress fibers and focal adhesions became thicker and larger after 20 h.

## Data Availability

The data presented in this study are available on request from the corresponding author.
